# Development and characterization of chitosan-polycarbophil interpolyelectrolyte complex-based 5-fluorouracil formulations for buccal, vaginal and rectal application

**DOI:** 10.1186/2008-2231-20-67

**Published:** 2012-10-25

**Authors:** Mohamed S Pendekal, Pramod K Tegginamat

**Affiliations:** 1 Department of Pharmaceutics, JSS College of Pharmacy, JSS University, SS Nagar, Mysore-15, Karnataka, India

**Keywords:** Chitosan, Polycarbophil, Interpolyelectrolyte complex, pH independent drug release

## Abstract

**Background of the study:**

The present investigation was designed with the intention to formulate versatile 5-fluorouracil (5-FU) matrix tablet that fulfills the therapeutic needs that are lacking in current cancer treatment and aimed at minimizing toxic effect, enhancing efficacy and increasing patient compliance. The manuscript presents the critical issues of 5-FU associate with cancer and surpasses issues by engineering novel 5-FU matrix tablets utilizing chitosan- polycarbophil interpolyelectrolyte complex (IPEC).

**Methods:**

Precipitation method is employed for preparation of chitosan and polycarbophil interpolyelectrolyte complex (IPEC) followed by characterization with Fourier transform infrared spectroscopy (FT-IR), Differential Scanning calorimeter (DSC) and X-ray Diffraction (XRD). 5-FU tablets were prepared by direct compression using IPEC. Six formulations were prepared with IPEC alone and in combination with chitosan, polycarbophil and Sodium deoxycholate. The formulations were tested for drug content, hardness, friability, weight variation, thickness, swelling studies, *in vitro* drug release (buccal, vaginal and rectal pH), *ex vivo* permeation studies, mucoadhesive strength and *in vivo* studies.

**Results:**

FT-IR studies represent the change in spectra for the IPEC than single polymers.DSC study represents the different thermo gram for chitosan, polycarbophil and IPEC whereas in X-ray diffraction, crystal size alteration was observed. Formulations containing IPEC showed pH independent controlled 5-FU without an initial burst release effect in buccal, vaginal and rectal pH. Furthermore, F4 formulations showed controlled release 5-FU with highest bioadhesive property and satisfactory residence in both buccal and vaginal cavity of rabbit. 3% of SDC in formulation F6 exhibited maximum permeation of 5-FU.

**Conclusion:**

The suitable combination of IPEC, chitosan and polycarbophil demonstrated potential candidate for controlled release of 5-FU in buccal, vaginal and rectal pH with optimum swelling approaching zero order release.

## Introduction

Extensive research has been made for the delivery of active drugs through buccal, vaginal and rectal cavity. The mucosa offers excellent opportunities to deliver active drugs both locally and systemically [1]. Over the last decade considerable progress in research towards the development of buccal, vaginal and rectal drug delivery for various diseases have been made.

Cancer is a disease involving all organs, races, ages and sex of the humans. Among all types of cancer, Oropharyngeal cancer, colorectal cancer contributes to world most burden and cervical cancer accounts for little extent. Oropharyngeal cancer develops in the part of the throat just behind the mouth called the oropharynx. Oropharynx includes the base of the tongue, soft palate, tonsils, tonsillar pillars and back wall of the throat [2]. Cervical cancer involving the cervix that is narrow and lower part of uterus. About 90% of Oropharyngeal and cervical cancers are squamous cells carcinoma that begins in the epithelial lining in mouth and vagina. Whereas in colorectal cancer columnar cells are affected. Surgery, radiation therapy and chemotherapy are the most common therapies for treating cancer.

In chemotherapy, most of the anticancer drugs are parenterally administered attributable to poor oral bioavailability; this can be due to either poor absorption or significant first pass metabolism. However, limitation of parental are need to be sterile, time consuming, pain on administration and as also studies reported, approximately 90% of cancer patients prefer oral administration than intravenous administration [3,4].

5-FU is the drug of choice in oropharyngeal caner, colorectal cancer, stomach cancer and cervical cancer [5]. Chemically, 5-FU is a dipodic acid and highly polar in nature with pka values of 8.0 and 13.0 [6,7]. After oral administration, 5-FU is poorly absorbed with erratic variation in bioavailability ranging between 0 to 80%. 5-FU after parenteral administration it is rapidly eliminated with apparent terminal half life of approximately 8-20 min [8,9]. On intravenous administration 5-FU produces severe systemic toxic effects including gastrointestinal, hematological, neural, cardiac and dermatological origin [10]. These problems make 5-FU suitable candidate for Transbuccal/vaginal and rectal delivery.

Polymeric drug delivery systems are mainly designed for the efficient delivery of active drug. Among various polymeric drug delivery systems, interpolyelectrolyte complexes are most new, efficient form of polymeric carriers for novel drug delivery systems [11-14]. Chitosan polycarbophil IPEC has established its potential in drug delivery and have been most efficient form of IPEC compared to other complexes [15-17].

Most publications on 5-FU focused only on single drug delivery system like buccal gels, cervical patches, colorectal drug delivery. Hardly any articles reported on permeation studies and histological effects of 5-FU. Literature review revealed that there is no single drug delivery system available that can be given either through buccal, vaginal or rectal route. The prime goal has to design 5-FU multipurpose tablets with greater efficacy, potency, adoptability to need, minimal toxic effects and better patient compliance than the established marketed product.

## Materials and methods

### Materials

5-FU obtained from Strides Arcolab Ltd., Bangalore, India. Chitosan (Marine chemical, Cochin, India, Deacetylation degree: 85%, Viscosity: <200 mpa.s, Moisture content: <10%, Ash content: <1%, Insoluble: <1%, pH: 3-6, Particle size: 80-100 mesh). Polycarbophil (Noveon AA-1)(Arihantt Trading Co., Mumbai, India, nature: pH:2.5-3, Ash content: 0.009 ppm, Density: (bulk) 0.19-0.24 g/cm^3^. Equilibrium moisture content: 8-10%, pka: 6.0 ± 0.5, Glass transition temp: 100-105°C, Moisture content: 2.0% max, Specific gravity: 1.41).

Sodium deoxycholate, microcrystalline cellulose and Talc were from Zydus Cadila, India. All other chemicals and reagents used were of analytical grade.

### Methods

#### Preparation of chitosan- polycarbophil complex (IPEC)

3% w/v Chitosan aqueous acetic acid solution and 3% w/v of polycarbophil acetic acid solution were mixed. The chitosan solution was added slowly to the polycarbophil solution under homogenization over a period of 20 min (5000 rpm) and the mixture was then stirred for a period of 1 hr at a speed of 1200 rpm with a mechanical stirrer. The formed gel was separated under vacuum pump and washed several times with a 2% v/v acetic acid solution to remove any non complexed polymeric material. The gel was dried in hot air oven and the dried complex was ground with a grinder. The powder was passed through a 200 μm sieve and used for further study.

#### Fourier transform infrared (FT-IR) spectroscopy study

The infrared absorption spectra of Chitosan, polycarbophil and IPEC were analyzed using a FT-IR spectrophotometer (8400S, Shimadzu, Japan). The pellets were prepared by pressing the sample with potassium bromide.

#### Differential scanning calorimetric (DSC)

Thermal analysis was carried out using a differential scanning calorimeter (DSC 50, Shimadzu Scientific Instruments, Japan) for Chitosan, polycarbophil and IPEC. The samples were placed in an aluminum-sealed pan and preheated to 200°C. The sample was cooled to room temperature and then reheated from 40 to 400°C at a scanning rate of 10°C/min.

#### Powder X-Ray diffraction

Powder x-ray diffraction patterns on Chitosan, Polycarbophil and IPEC were obtained by using an x-ray Diffractometer (Miniflex II Desktop X-ray Diffractometer, Rigaku Corporation, Tokyo, Japan). The samples were scanned from 6° to 40° (2θ) with an increment of 0.02° and measurement time of 10 s/increment.

#### Preparation of mucoadhesive matrix tablet

Mucoadhesive tablets were fabricated by direct compression method as shown Table 1. The accurate quantity of 5-FU and excipients were weighed. They were passed through sieve and thoroughly mixed using mortar and Pestle. The blend was lubricated and then compressed into compacts by direct compression method using 8-mm flat-faced punches in KBr press (Techno search, Mumbai, India) at 1 ton pressure with a dwell time of 1 s.

**Table 1:** Formulation chart

**Ingredients**	**F1**	**F2**	**F3**	**F4**	**F5**	**F6**
5-flurouracil	20	20	20	20	20	20
IPEC	60	80	100	80	80	80
Chitosan	----	----	----	20	20	20
Polycarbophil	----	----	----	20	20	20
Sodium deoxycholate	----	----	----	---	3	4.5
Microcrystalline cellulose	65	45	25	5	2	0.5
Talc	5	5	5	5	5	5

Weight in mg.

Total weight of tablet is 150 mg.

#### Swelling studies

The swelling index of the prepared mucoadhesive 5-FU tablets was determined by weighing five tablets and recording their weights before placing them separately in weighed beakers. The total weight was recorded (*W*1). Four milliliters of phosphate buffer pH 6.8 (similarly with simulated vaginal fluid pH 4.2 and pH 7.4) was added to each beaker and then placed in an incubator at 37 ± 0.5°C. At time intervals of 2, 4, 6 and 8 h excess water was carefully removed, and the swollen tablets were weighed (*W*2). The experiment was repeated three times, and the average *W*1 and *W*2 were reported.

The swelling index was determined from the formula.

$$SI=\left(\text{W2-W}1\right)\text{/W}1\;\text{X}\;100$$

#### *In vitro* release of matrix tablets

The drug release rate from buccal compacts was studied using the orbital shaking incubator using (Remi CIS 24, India) 30 mL of phosphate buffer pH 6.8. The temperature was maintained at 37 ± 0.5°C and 50 rpm (rotation per min). For every one hour of time interval 3 mL sample was withdrawn, filtered through a Millipore filter of 0.45 μm pore size and assayed spectrophotometrically at 266 nm. Immediately after each sample withdrawal, a similar volume of phosphate buffer pH 6.8 was added to the dissolution medium.

The drug release rates from vaginal tablets were studied in 500 ml of simulated vaginal fluid pH 4.2 in type II dissolution apparatus. The temperature was maintained at 37 ± 0.5°C and 50 rpm. For every one hour of time interval 10 mL sample was withdrawn, filtered through a Millipore filter of 0.45 μm pore size and assayed spectrophotometrically at 265 nm. Immediately after each sample withdrawal, a similar volume of simulated vaginal fluid was added to the dissolution medium.

*In-vitro* drug release for rectal tablets was performed using the dissolution apparatus I; 500 mL phosphate buffer (pH 7.4) maintained at 37 ± 0.5°C was used as a dissolution medium. Basket was rotated at 50 rpm. 10 mL aliquots was taken at periodic time intervals and replaced by equal volume of phosphate buffer. The solution was suitably diluted and the absorbance was taken at 267 nm using UV visible Spectrophotometer.

#### Bioadhesive strength

Bioadhesive strength of the compacts was measure using modified physical balance as recently discussed [18]. *In vitro* bioadhesion studies were carried out using sheep buccal mucosa and modified two-armed balance. The phosphate buffer pH 6.8 was used as the moistening fluid. A glass stopper was suspended by a fixed length of thread on one side of the balance and was counter balanced with the weights on the other side. Fresh sheep buccal mucosa was collected from the slaughter house. It was scrapped off from the connective tissues and a thin layer of buccal mucosa was separated which was stored in tries buffer until used for the bioadhesion study. A circular piece of sheep buccal mucosa was cut and fixed to the tissue holder and was immersed in phosphate buffer pH 6.8 and the temperature was maintained at 37° ± 1°C. Then the tablet was fixed to a glass stopper with the help of cyanoacrylate adhesive and it was placed on the buccal mucosa by using a preload of 50gm and kept it aside for 3 min to facilitate adhesion bonding. After preloading time, the preload was removed and the weights were added on the other side of the balance until tablet detaches from the sheep buccal mucosa. The weight required to detach tablet from buccal mucosa was noted.

#### *Ex vivo* permeation study

Permeation study was carried out for the optimized formulation using Franz diffusion cell. The tablet was placed in the donor compartment on the sheep mucosa. The mucosal layer is on donor compartment. The receptor compartment was filled with phosphate buffer pH 6.8. The temperature was maintained at 37 ± 0.5°C and 50 rpm. The amount of 5-FU permeated through sheep mucosa was determined by withdrawing 3 ml of aliquots from the receptor compartment using a syringe and immediately replacing the same volume of solution.

#### *In vivo* x-ray studies

The *in vivo* X-ray studies were approved by the Institutional Animal Ethical Committee of JSS College of Pharmacy (Mysore, Karnataka, India). The study was performed on healthy female rabbit, weighing between 1-1.5 kg. F4 formulation was modified by adding 20 mg of X-ray grade barium sulfate (20 mg of 5-FU was replaced). The prepared tablet was placed in buccal mucosa of healthy rabbit. During the study, the rabbit was not allowed to eat or drink. The rabbit was exposed to x-ray examinations and photographs were taken at 1^st^ and 8^th^ hr after administration of the tablet. Similarly procedure was followed for vaginal and rectal delivery.

#### Kinetic analysis

Drug release from simple swellable systems may be described by the power law expression and is defined by the following equation

$$M_{t}/M_{\infty }={\text{Kt}}^{n}$$

Where M_t_ is the amount of drug released at time t, M_∞_ is the overall amount of drug released, K_1_ is the release constant; n is the release or diffusion exponent and M_t_/M_∞_ is the cumulative drug concentration released at time t (or fractional drug release).

The release exponent (n) value was used for interpretation of the release mechanism from the compacts. The dissolution data were modeled by using PCP disso v2.01 (Bharathi Vidhyapeeth, Deemed University, Pune, Maharashtra, India).

#### Statistical analysis

Statistical analyses of all data were undertaken using Graph Pad prism version 5.0 (Graph pad software Inc, San Diego, California, USA).

## Results and discussion

In our previous paper, studies on Carbopol® 71 G and Noveon AA-1(polycarbophil) polymers and the influence of formulation expedients were evaluated in our laboratory on buccal bioadhesive tablet of Fluconazole [19]. Later, it was also shown in our laboratory that the interpolymer complex between Chitosan and Carbopol® 71 G as a suitable polymer for the development of novel drug delivery of miconazole for candidiasis [20].

In the light of vast previous experience and literature on Carbopol® 71 G and polycarbophil, we identified that polycarbophil, Carbopol and chitosan polymers are well suitable for particular pH. In contrast, chitosan-polycarbophil interpolyelectrolyte complex showed high potential as matrix former and used as new class of polymer carriers for creating novel drug delivery system [21]. Therefore based on the extensive review and previous experience on above cited polymers, chitosan-polycarbophil interpolyelectrolyte complex are taken into present investigation.

### Characterization of IPEC

**Figure 1:**
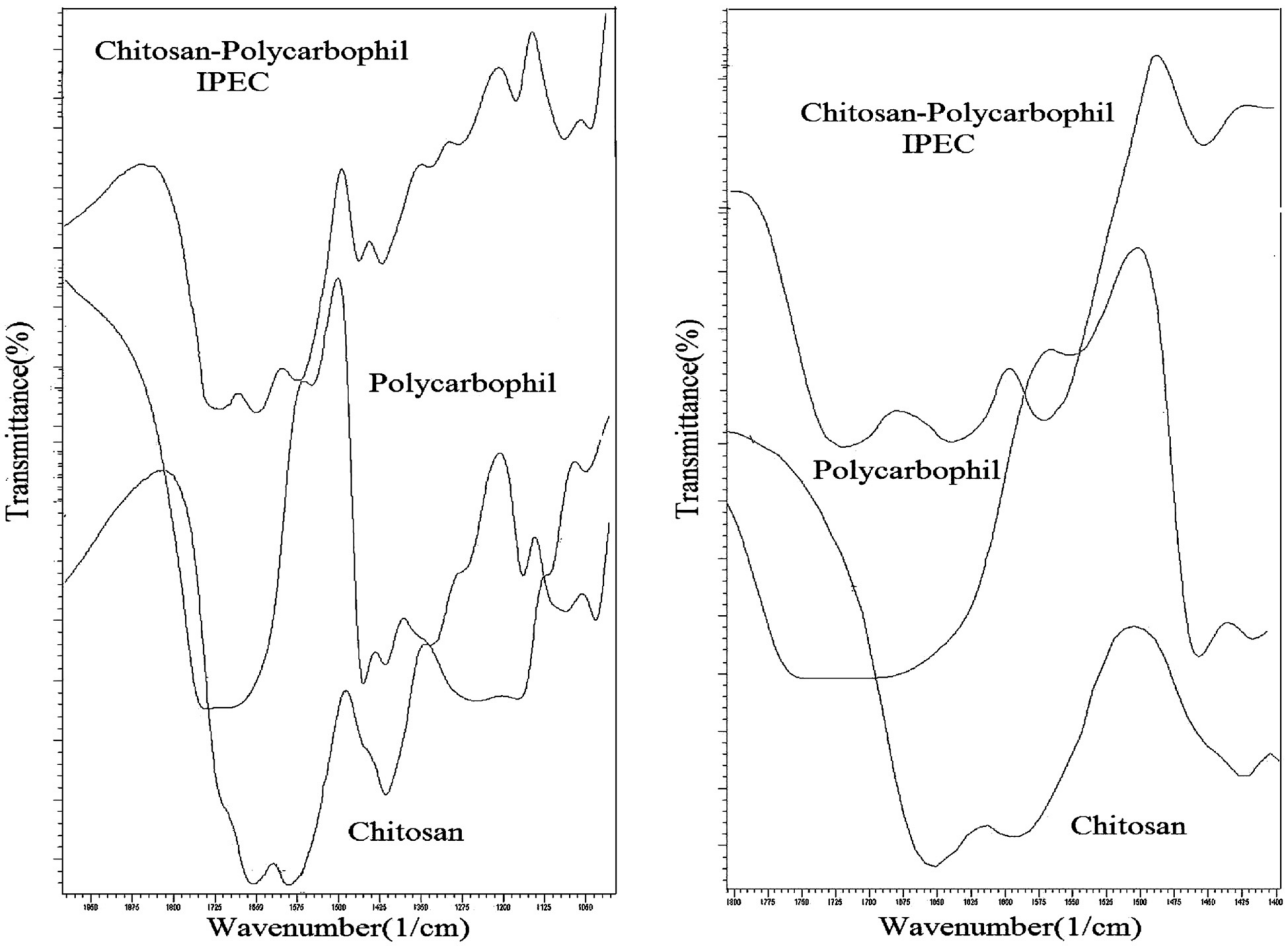
**FT-IR Spectra of Chitosan, Polycarbophil and IPC in 2000-1000 and 1800-1400 cm**^**-1**^.

The interaction between chitosan and polycarbophil has been studied by several investigators [22,23]. The studies indicated that IPEC could be formed by the electrostatic interaction between the COO^−^ group of polycarbophil and NH_3_^+^ group of chitosan. The protonation of chitosan and dissociation of polycarbophil solution was successfully accomplished by solution of chitosan and polycarbophil in acetic acid solution. Subsequently, chitosan –polycarbophil interpolymer complex were prepared from these solutions. Figure 1 shows the superimposed IR spectra of Chitosan, polycarbophil and IPEC in 1000-2000 cm^-1^ and 1400-1800 cm^-1^.

The degree of deacetylation of Chitosan is 85%, the amine group of 2-aminoglucose unit and the carbonyl group of 2-aminoglucose unit of chitosan showed absorption bands at 1589 and 1656 cm^-1^, respectively [24]. Polycarbophil exhibits a broad band at 1713 cm^-1^ assigned to C = O stretching (hydrogen-bonded). The weak band at 1415 cm^-1^ is due to the symmetric stretching of carboxylate anion (COO^−^), bands 1230 and 1160 cm^-1^ are attributed to the C-O stretching [25,26]. In IR spectra, spectra of the physical mixture of two polymers or immiscible polymers will be the sum of the spectra of the individual compounds, whereas polymers after electrostatic interaction, there will be changes in the IR spectra such as Wave number shifts, band broadening and new absorption bands that are evidence of the polymers miscibility [27]. In IR spectra of Chitosan-polycarbophil IPEC, a new and strong band is observed at 1561 cm^-1^. This band can be assigned due to the overlapping of asymmetric COO^-^ stretching vibration of polycarbophil and the NH_3_^+^ asymmetric bending vibration of chitosan which is agreement with literature to be located between 1550–1610 cm^-1^[28]. In addition, another band at approximately 1402 cm^−1^ is a further evidence of the interaction because it is attributed to the symmetric COO^−^ stretching vibration [29].

**Figure 2:**
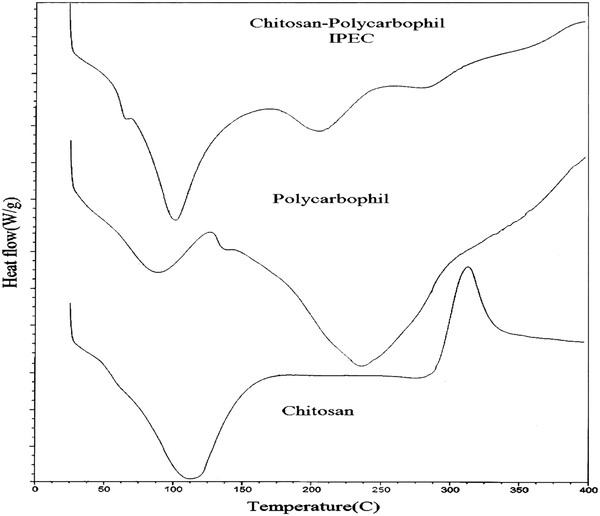
**Superimposed DSC thermograms of chitosan, polycarbophil and IPEC**.

Figure 2 shows the DSC thermo grams of chitosan, polycarbophil and IPEC. The DSC thermo grams of pure chitosan, exhibits one broad endothermic peak at 110°C associated to the evaporation of bound water, a glass transition at 240°C and an exothermic peak at about 320°C attributable to the polymer degradation. This includes saccharide rings dehydration, depolymerization and decomposition of deacetylated and acetylated chitosan units [30,31]. These peaks are agreement with other reported studies [32,33]. Polycarbophil thermo gram exhibits two endothermic peaks at 91°C and ~245°C. The first endothermic peak is short and narrow peak assigned to the evaporation of water from hydrophilic groups in the polymers and the second one corresponds to a thermal degradation through intermolecular anhydride formation and water elimination [34,35]. The chitosan-polycarbophil IPEC thermo gram exhibit four endothermic peaks. The first one and second is associated with the vaporization of water situated at ~53°C and ~100°C in the chitosan polycarbophil IPEC. The second endothermic peak is probably related with the cleavage of the electrostatic interactions between the oppositely charged polymers, since it is not observed for the pure compounds. The appearance of new broad endotherm at 250°C is indicative of a compound with distinctive thermal behavior properties.

**Figure 3:**
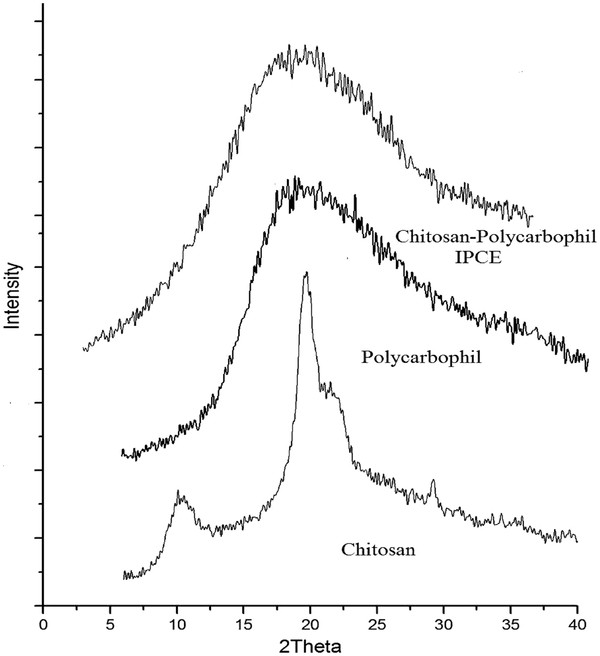
**Superimposed XRD spectra of Chitosan, polycarbophil and IPEC**.

The X-ray diffraction of Chitosan, polycarbophil and IPEC were shown in Figure 3. The powder x-ray diffraction pattern of chitosan powder showed two prominent diffraction peaks at 10.6° (2θ) and 19.64° (2θ). A shoulder peak appears at 21.74° and also minor peak appears at 26.62°. The two prominent crystalline peaks at 10.6° and 19.64° are typical fingerprint for chitosan which were related to the hydrated and anhydrous crystals respectively [36]. Polycarbophil showed peaks at 18.88°, whereas IPEC showed peak at 19.92°. The typical peaks of chitosan disappeared and the IPEC showed an amorphous morphology after completing. The integration of polycarbophil into chitosan disrupted the crystalline structure of chitosan, hindering the formation of hydrogen bonding between amino groups and hydroxyl groups.

### Characterization of matrix tablets

#### Pharmaceutical properties

The results of pharmaceutical properties are summarized in Table 2. The matrix tablets showed a diameter of 8 mm with negligible variation and therefore not included in the table. All the formulations showed satisfactory values within the limits of conventional oral tablets stated in the *Indian Pharmacopoeia*[37].

**Table 2:** Pharmaceutical properties

**Formulation code**	**Thickness (mm)**	**Hardness (N)**	**Friability (%)**	**Weight (mg)**	**Drug content (%)**
F1	2.03 ± 0.01	71 ± 3	0.08	149.4 ± 0.43	99.90 ± 0.09
F2	2.01 ± 0.05	72 ± 2	0.09	149.2 ± 1.00	99.91 ± 0.08
F3	2.03 ± 0.01	72 ± 3	0.06	150.0 ± 0.57	99.92 ± 0.05
F4	2.03 ± 0.02	73 ± 1	0.05	150.1 ± 1.00	99.94 ± 0.08
F5	2.01 ± 0.02	73 ± 3	0.03	149.2 ± 0.52	99.95 ± 0.05
F6	2.02 ± 0.02	73 ± 2	0.04	149.2 ± 0.44	99.92 ± 0.05

#### Swelling studies

**Figure 4:**
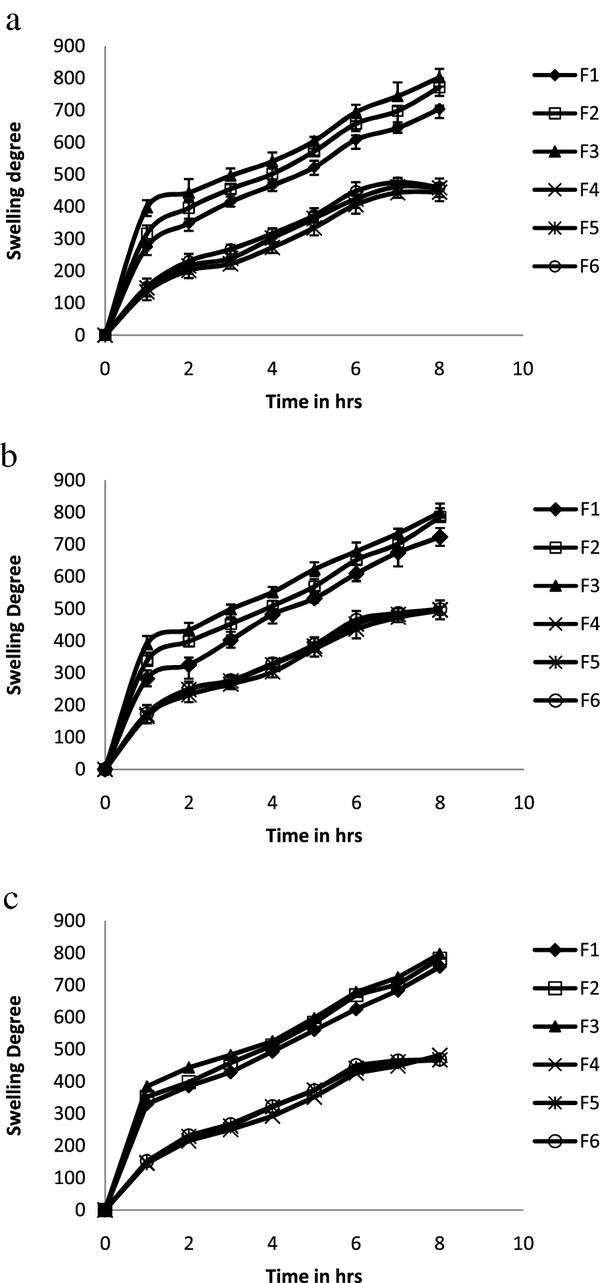
**a: Swelling studies of formulations in pH 4.2.b**: Swelling studies of formulations in pH 6.8. **c**: Swelling studies of formulations in pH 7.4.

F3 formulation containing higher concentration of IPEC showed pronouncedly higher swelling capabilities in vaginal, buccal and rectal pH compare to F1 and F2 formulation (lesser concentration of IPEC). The swelling for F3 formulation in vaginal, buccal and rectal pH was found to 802%, 798%, and 797% respectively. Further, the addition of chitosan and polycarbophil for F4 formulation in vaginal pH 4.2 reduces the swelling to 445%. Nearly same degree of swelling was observed for F5 and F6 as that of the formulation F4. The addition of sodium deoxycholate doesn’t significantly alter the swelling in F5 and F6. The comparison of degree of swelling of all formulations in vaginal pH 4.2 was shown in Figure 4a. In pH 6.8, F2 formulation showed 785% of swelling i.e. lesser than F3 formulation. The F4 formulation found to be having 495% of least swelling; this may be explained due to dissociation of carboxylic group of polycarbophil in buccal pH 6.8. Swelling profiles of formulations in pH 6.8 was shown in Figure 4b. In pH 7.4, F4 formulation exhibited swelling of 481% that is as nearly similar in pH 4.2 and pH 6.8. Nearly same degree of swelling was observed for F5 and F6 as that of the formulation F4. Swelling profiles of formulations in pH 7.4 was shown in Figure 4c.

These findings indicate the presence of IPEC alone in matrix tablet exhibit slow uniform pH independent swelling degree and also the presence of chitosan and polycarbophil in IPEC matrix tablets alters the swelling degree. Therefore the mechanism of drug release from IPEC matrix tablets was affected by the presence of chitosan and polycarbophil.

#### Bioadhesion studies

**Figure 5:**
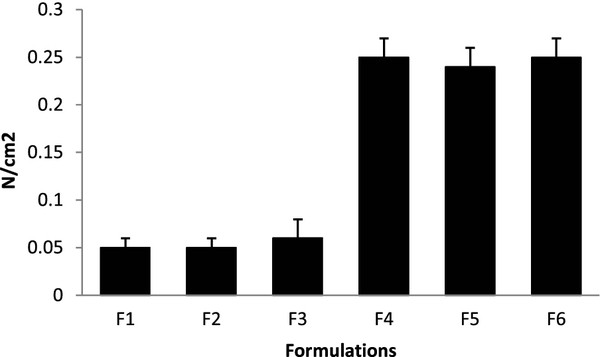
**Mucoadhesive strength of formulations**.

The mucoadhesive strength for all the formulations was shown in Figure 5. F1 formulation containing only IPEC shows least detachment force this may be explained due to the lacking of free functional groups which probably involved in the adhesion of the mucosa. F2 formulation having higher concentration of IPEC still exhibited least detachment force, even further increase in IPEC concentration in F3 formulation doesn’t alters the detachment force. F4 formulation shows highest detachment force, this may be due to availability of free functional groups. F5 & F6 formulation containing sodium deoxycholate doesn’t have any impact on the detachment force.

These findings indicate the presence of IPEC alone doesn’t exhibit sufficient bioadhesion hence the presence of other polymers is necessary for development of bioadhesive matrix tablets. Therefore suitable combination of polymers along with IPEC is selected for producing sufficient bioadhesion without altering properties of IPEC.

#### *In vitro* drug release studies

**Figure 6:**
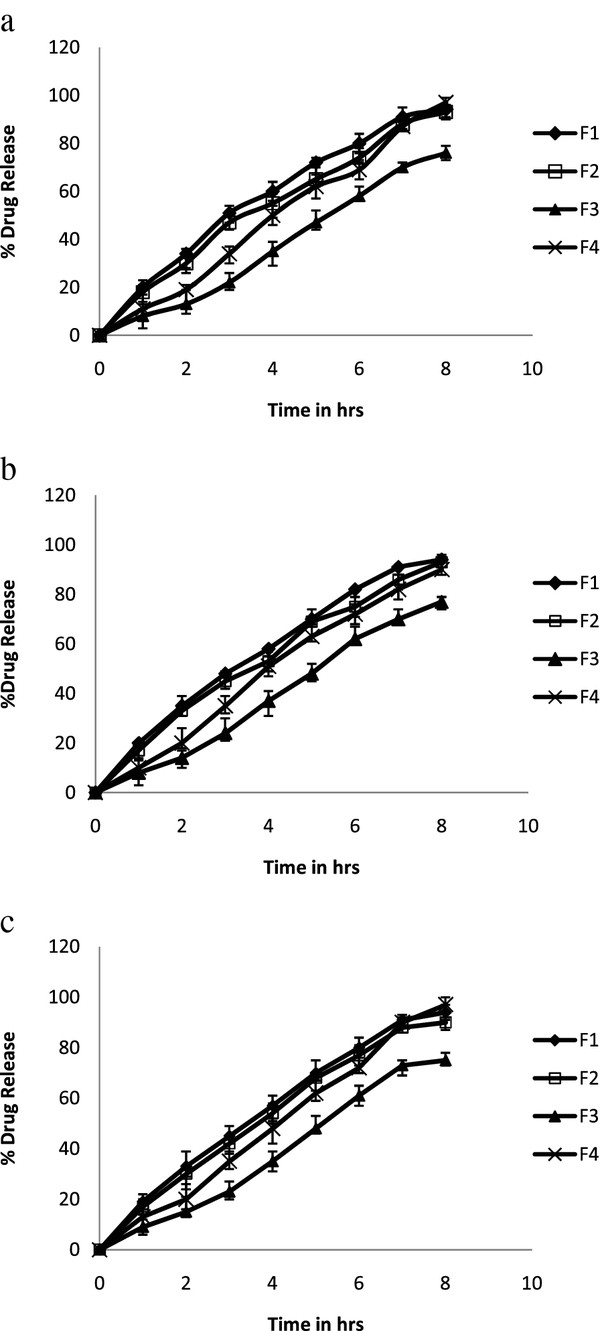
**a: Dissolution profile of formulations F1-F4 in pH 4.2.b**: Dissolution profile of formulations F1-F4 in pH 6.8. **c**: Dissolution profile of formulations F1-F4 in pH 7.4.

In vitro drug release study of formulations in pH 4.2, 6.8 and 7.4 was shown in Figure 6a, b and c respectively. Initially 5-FU matrix tablets were made from IPEC and their *in vitro* drug release investigated and exhibited controlled release properties without any initial burst effect in buccal, vaginal and rectal pH .F1 & F2 formulations exhibited controlled drug release in buccal, vaginal and rectal pH with zero-order drug release. But F3 formulation, retarded drug release up to 70% in buccal, vaginal and rectal pH. Higher increase in concentration of IPEC forms gel layer around tablets that retards the drug release up to 70%. In spite of excellent controlled 5-FU release up to 8 hr in buccal, vaginal and rectal pH, formulations containing IPEC (F1, F2 & F3) fails to attain sufficient mucoadhesive strength (mentioned in mucoadhesive studies). The study represents that alone IPEC can sustain the drug release but doesn’t produces mucoadhesion. Hence, addition of chitosan and polycarbophil for IPEC formulation exhibited drug release up to 90% in 8 h in buccal, vaginal and rectal pH. This may be due to undissociation of carboxylic group of polycarbophil in pH 4.2, thereby neutralizing the gel forming ability of chitosan. In buccal pH 6.8, the polycarbophil has the ability to swell and retard the drug release that is due to dissociation of carboxylic group. This property of polycarbophil is neutralized by chitosan that possesses gel forming property only in acidic pH. Similarly in rectal pH 7.4 the properties of polycarbophil and chitosan don’t altered, hence similar drug profile was seen as in buccal and vaginal pH. F5 & F6 formulation containing SDC exhibited similar drug release profile as F4 formulation (Data not mentioned).

F4 formulation was subjected for kinetic study and the Correlation coefficients for F4 formulation in vaginal, buccal and rectal pH were found to 0.9964, 0.9957 and 0.9966 respectively. According to drug release exponent value (n), it is clear that F4 formulation approached zero-order drug release in vaginal, buccal and rectal pH (n value is 0.9). Thus the drug release is predicted as function of swelling for F4 formulation.

To confirm the similarity of F4 formulation dissolution profiles in buccal, vaginal and rectal pH, the similarity factor (f2) was used and was found above 80. Since the f2 values were higher than 50, these results confirmed that the drug release profiles were almost similar for F4 formulation for both buccal, vaginal pH and rectal pH.

#### *Ex vivo* permeation studies

**Figure 7:**
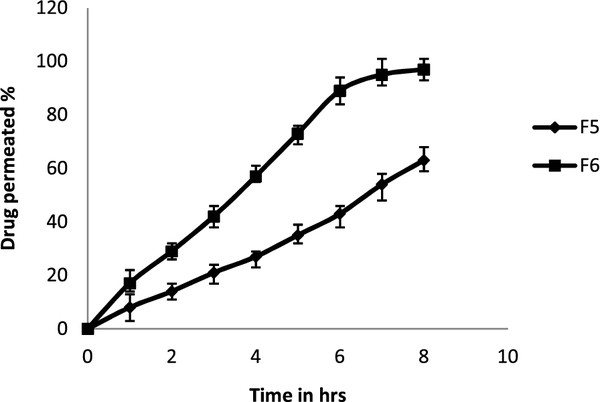
**Ex vivo permeation studies of F5 & F6 formulation**.

5-FU permeation from formulations F5 and F6 across sheep mucosa over a period of 8 h is shown in Figure 7. The maximum permeation of 5-FU from F5 was 97% at 8 h compared with 63% from F6. Regression of the linear portions of the two plots gave Slopes and intercepts from which the permeation flux (slope divided by mucosal surface area) of F5 and F6 were calculated to be 8.5866 and 5.1333 mg/cm^2^/h, respectively. While permeation coefficients were found be 2.1466 and 1.2833 cm/h for F5 and F6 formulations, respectively.

In formulation F5 addition of SDC 2% increased the cumulative percentage of drug permeated to 63%. This may be due to SDC extracted only mucosal lipid from the intercellular spaces. Thus, this enhances the diffusivity of the 5-FU via the par cellular or polar route. Further increase in concentration of SDC (F6),i.e., 3%, increased the drug permeation up to 97% thus SDC in 3% extract lipids from the cell membranes, along with the extraction of mucosal lipid from the intercellular spaces by the formation of micelles. This resulted in enhancing passive diffusivity of the 5-FU via transcellular (crossing the cell membranes and entering the cell) and par cellular routes [38]. It was mentioned that SDC can also cause the uncoiling and extension of the protein helices, which leads to opening of the polar pathways for diffusion [39]. All these effects might contribute to enhancing the permeation of the drug.

#### *In vivo* X-ray studies

**Figure 8:**
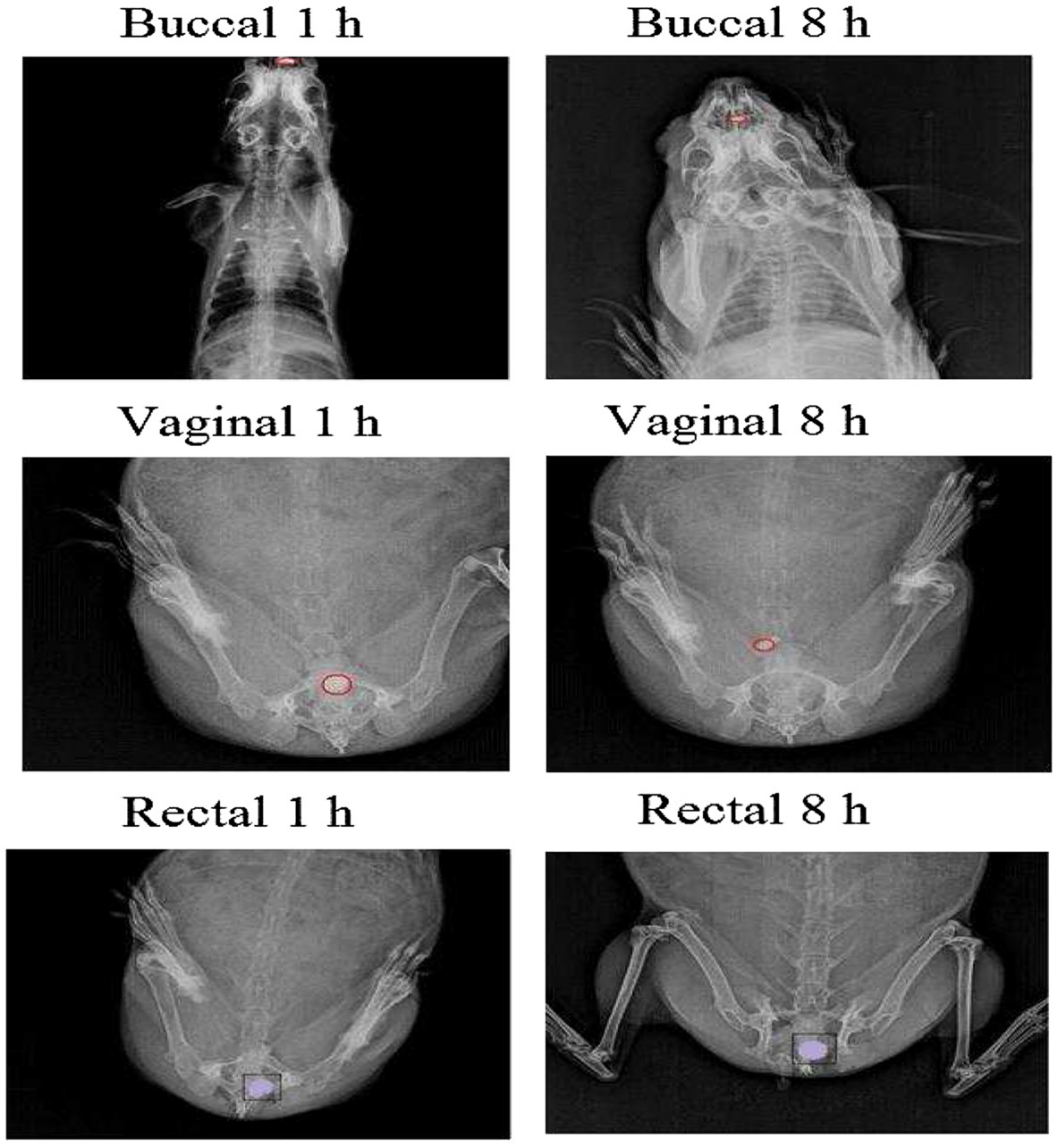
**X-ray radiographic images of buccal and vaginal cavity at 1 and 8 h after ingestion of BaSO**_**4**_**-loaded optimized F4 matrix tablet**.

After administration of the optimized formulation (F4), developed by using barium sulfate, the duration of the tablet in the buccal, vaginal and rectal cavity was monitored by radiograms (Figure 8). The tablet adheres to the buccal, vaginal and rectal mucosa. The buccal tablet swells and retained till 8 h with little reduction in tablet size, as the tablet swells certain part of the tablet was swallowed by rabbit thereby the tablet size reduces. Whereas the tablet in vaginal cavity swells in 1 h and little reduction of size at 8 h. In rectal cavity tablet remained in cavity till 8 h.

## Conclusion

All formulations studied showed good pharmaceutical properties. However, in consideration of swelling, drug release and mucoadhesion, some important differences have been highlighted. Formulation F3 containing only IPEC exhibited almost similar swelling in vaginal, buccal and rectal pH. Other two formulations F1 & F2 also containing lesser amount of IPEC produced less swelling than F3 formulation. The addition of polymer blends (chitosan and polycarbophil) to F3 formulation showed optimum swelling. These formulations, in fact, in addition to good swelling, lack sufficient mucoadhesion.F4 formulation demonstrated high mucoadhesive property. Further, the addition of SDC doesn’t produce impact on mucoadhesion.

On the basis of these properties, it is clear that IPEC alone doesn’t produce desired response hence, addition of polymeric blends is essential for developing the drug delivery system. The *in vitro* drug release study of F3 formulation demonstrated potential excipient for control release and also possesses pH independent drug release. The matrix system with suitable combination of IPEC with polymeric blends (chitosan and polycarbophil) in F4 formulation able to produce desired drug release, bioadhesion, swelling and satisfactory *in vivo* residence. The desired bioadhesion can be achieved only with the addition of chitosan and polycarbophil; hence suitable combination played key role in the bioadhesion and subsequently maintains the pH independent drug release without initial burst release pattern. The addition of 3% Sodium deoxycholate to the F4 formulation demonstrated necessary 5-FU permeation.

## Competing interests

Both authors declared that they have no competing interests.

## Authors' contributions

MSP carried out preparation and characterization of chitosan-polycarbophil complex, involved in formulation of tablets, carried out physicochemical properties, swelling studies, bioadhesion studies, kinetic studies and statistical analysis. PKT carried out *in vitro* drug release studies, *in vivo* studies and has been involved in drafting the manuscript. Both authors read and approved the final manuscript.
